# Field study to investigate the effectiveness and safety of a novel orally administered combination drug product containing milbemycin oxime and lotilaner (Credelio^®^ Plus) for the prevention of heartworm disease (*Dirofilaria immitis*) in client-owned dogs in the USA

**DOI:** 10.1186/s13071-021-04767-6

**Published:** 2021-05-28

**Authors:** Lisa M. Young, Scott Wiseman, Elizabeth Crawley, Kim Wallace, Daniel E. Snyder

**Affiliations:** 1 Elanco Animal Health Research and Development, 2500 Innovation Way, Greenfield, IN 46140 USA; 2 Elanco Animal Health, Form 2, Bartley Way, Bartley Wood Business Park, Hook, RG27 9XA Hants UK; 3 Daniel E. Snyder, DVM PhD. Consulting, LLC, Indianapolis, IN 46229 USA

**Keywords:** *Dirofilaria immitis*, Heartworm, Prevention, Macrocyclic Lactone, Milbemycin oxime, Lotilaner, Field study, Dog

## Abstract

**Background:**

*Dirofilaria immitis,* a globally distributed filarial parasite of dogs, is known to cause serious or fatal cardiopulmonary disease. Client-owned dogs were enrolled in a clinical field study in the USA to evaluate the clinical effectiveness and field safety of an orally administered combination investigational product (IP) containing milbemycin oxime and lotilaner (Credelio^®^ Plus) as compared to a control product (CP) for the prevention of heartworm disease when administered monthly for 11 consecutive months.

**Methods:**

In this 11-month field study, 319 dogs ≥ 8 weeks old confirmed to be heartworm-negative were enrolled from eight geographically distinct US veterinary clinics, including sites in the southern USA and Mississippi River Valley. The dogs were treated with either the IP combination product at 0.75–1.53 mg/kg milbemycin oxime and 20–41.5 mg/kg lotilaner (*n* = 159) or the CP (Sentinel^®^ Flavor Tabs^®^; milbemycin oxime/lufenuron) at the label-recommended dose rate (*n* = 158.) On day 330, effectiveness was evaluated in each dog using antigen and microfilarial (modified Knott’s) testing to assess the establishment of any patent adult heartworm infections.

**Results:**

All dogs treated with the IP combination product and the CP tested negative (100% prevention) for heartworm infection on day 330. The IP combination product tablets containing milbemycin oxime and lotilaner were well tolerated based on the safety assessments in all treated dogs.

**Conclusions:**

This multi-site clinical study using client-owned dogs demonstrated that monthly use of flavored, chewable tablets containing a combination of milbemycin oxime and lotilaner administered orally under end use conditions is safe for dogs. None of the enrolled dogs developed heartworm infections. Eleven consecutive monthly treatments of the IP provided 100% prevention of heartworm disease caused by *D. immitis*.

**Graphic Abstract:**

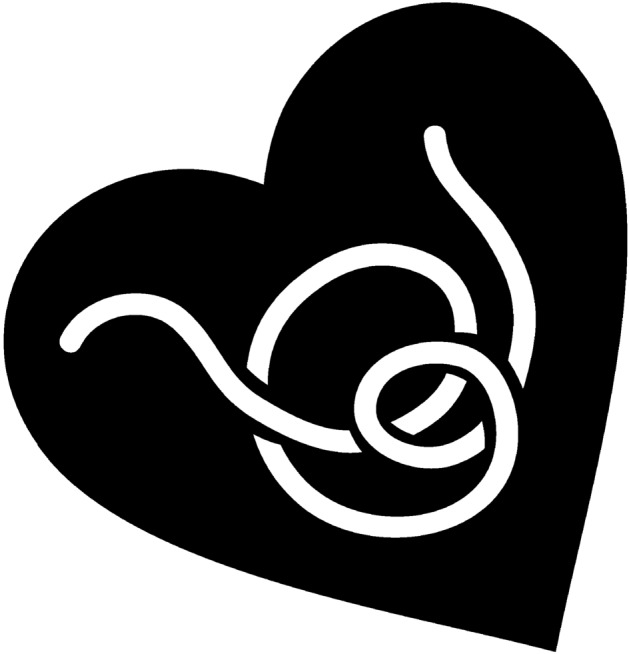

## Background

Heartworm (*Dirofilaria immitis*), a globally distributed filarial parasite in dogs and other animals, is transmitted by several different mosquito species and is known to cause significant cardiopulmonary disease [1, 2]. Historically, heartworm (HW) disease has been prevented in dogs by prophylactic treatment with a number of approved oral, topical or injectable macrocyclic lactone (ML) drug products [2, 3]. The American Heartworm Society (AHS) recommends year-round administration of these preventive products due to the severity of HW disease, and other global scientific organizations have recommendations for the control of this serious disease [4–7]. Over the last several years there have been reports of lack of effectiveness (LOE) of all approved ML containing drug products that are available as a HW preventive [3, 8–10]. The causes of these LOEs are likely multifactorial in nature. These causes have been documented to include poor owner compliance, incorrect dose rate based on the body weight of the dog, missed doses, incorrect interpretation of HW diagnostic tests, documented cases of HW resistant to ML, and improvements in the sensitivity and specificity of adult HW antigen tests [11]. Research indicates that prophylactic HW prevention effectiveness may also depend on the active ingredient in the formulation, the dose level, and the dosage regimen that is utilized [12].

Milbemycin oxime (MO) is a ML that was originally developed to treat adult intestinal nematode infections in dogs and also used for monthly HW prevention when dosed orally at a minimum effective dosage of 0.5 mg/kg [13, 14]. The safety of MO in dogs with patent HW infections with circulating microfilariae also has been demonstrated [15]. The dose limiting intestinal nematode species for MO at 0.5 mg/kg was shown to be the adult stage of hookworm, *Ancylostoma caninum* [16]. To effectively kill larval and immature adult stages of *A. caninum* and *Toxocara canis*, it was subsequently demonstrated that the required minimum oral dose of MO was 0.75 mg/kg [17].

In order to provide broad-spectrum adult and additionally larval endoparasite effectiveness as well as flea, mite and tick treatment, prevention, and control in dogs, MO at a minimum dosage of 0.75 mg/kg was selected for inclusion in an oral chewable tablet formulation in combination with lotilaner (isoxazoline class of chemistry). Tablets of this combination drug product contain a minimum dosage of 0.75 mg/ kg (range, 0.75–1.53 mg/kg) of MO and a minimum dosage of 20 mg/kg (range, 20–41.5 mg/kg) of lotilaner.

Lotilaner is an ectoparasiticide and is from a newer class of chemistry called the isoxazolines. Lotilaner was previously developed as a monthly, orally administered chewable tablet for use in dogs and cats as a mono-use drug product (Credelio^T.M.^, Elanco Animal Health). Credelio has been shown to provide fast and consistent month-long effectiveness against fleas and ticks in dogs and cats [18–20]. It was also shown to have effectiveness against *Demodex* sp. mites in dogs [21].

Global veterinary practice guidelines (e.g. AHS, ESCCAP, CAPC) recommend quarterly or year-round prevention, control, and/or treatment for HW and lungworms such as *Angiostrongylus vasorum*, certain intestinal nematode parasites, and fleas, ticks and mites that commonly infect/infest dogs [4–7]. Additionally, a broad-spectrum drug product that can reduce the transmission of zoonotic pathogens and zoonotic parasites and prevent blood-sucking flea and tick infestations can provide a significant clinical health benefit for dog owners and their pets. With this desire to provide a novel broad-spectrum oral chewable tablet combination formulation that includes MO and lotilaner, this new drug product was developed and assessed for prevention of HW and lungworm (*A. vasorum*) disease, to control flea and tick infestations for 1 month, and to treat and control intestinal roundworms (*T. canis*, *Toxascaris leonina)*, hookworms (*A. caninum*), and whipworms (*Trichuris vulpis*) that are common in dogs.

Data as summarized within this publication were obtained in order to evaluate the effectiveness and safety of this novel MO and lotilaner combination investigational product (IP) administered monthly as an oral chewable tablet (Credelio Plus^®^) as compared to an approved control product (CP) for the prevention of HW disease in client-owned dogs under end-use clinical field conditions.

## Methods

### Field study

This US-based clinical field study utilized a total of eight geographically diverse veterinary clinics. Selected clinic locations in the eastern half of the USA included sites in the southern and southeastern USA and Mississippi River Valley. The clinic sites were selected in order to provide maximum exposure to HW infections in a real-world setting. This was achieved by enrolling adequate numbers of dogs into each IP and CP treatment group and by choosing areas of the USA where HW is endemic as demonstrated by prevalence data generated by CAPC. The field study was conducted in selected sites within peak periods during HW transmission months. The CAPC reported that for six of the eight study sites (by county level) where the animals were located, local prevalence varied from 1.56 to 6.34%, which is above the national prevalence for *D. immitis* (1.28%). No single clinic represented more than 40% of the evaluable cases for product effectiveness or safety assessments. For inclusion in the respective product effectiveness evaluations, a clinic was required to have a minimum of five evaluable cases involving at least two evaluable cases from each treatment group. The study design was a randomized, single-masked, multi-center clinical study using the CP, Sentinel^®^ Flavor Tabs^®^ (CP: milbemycin oxime/lufenuron; Merck) as compared to the IP.

Use of the MO and lotilaner combination IP in this study was intended to evaluate a proposed commercial formulation and dose regimen under end-user conditions for the intended label indications in dogs. This study was conducted to partially fulfill the requirements for demonstrating substantial evidence of product effectiveness and safety in the interest of approval for marketing as required by global regulatory agencies. Additionally, the study complied with Good Clinical Practice (GCP) guidelines [22].

### Animals

The study was a multi-site clinical study involving client-owned dogs under field conditions. To be enrolled in the study, on day −1 the owner was required to complete and sign an owner consent form and to provide the dog’s prior and current medical history. In multi-dog households, only one dog per household was screened and enrolled. The individual dog was considered the experimental unit, and product effectiveness was evaluated for each dog that completed the study. All dogs treated with the correct IP or CP product were evaluated for product safety.

The dogs were recruited by the eight veterinary clinics from existing and referred client households. Dogs considered for enrollment had to be at least 8 weeks of age and weigh ≥ 2.0 kg on day −1. Dogs of any breed and sex, reproductively neutered or intact (non-pregnant and non-lactating if female), and not intended for breeding during the study were eligible for enrollment. Examples of other animal eligibility criteria included the following: the dog was of a suitable temperament (not fractious); within 365 days before visit 1, the dog had not been treated with a long-acting injectable HW preventive containing moxidectin; the dog was generally healthy, i.e., expected to survive the 11-month study duration based on history and physical examination; the dog was free of a serious disease that would interfere with the objectives of the study; and the dog had received a monthly HW preventive medication for ≥ 2 consecutive months before visit 1, the most recent treatment having been given within 21–30 days before visit 1. Dogs ≥ 6 months of age were required to have a negative adult HW antigen test and no circulating microfilariae (including *Dirofilaria* sp. and *Acanthocheilonema* sp.).

### Design

A randomization algorithm within the electronic data capture system employed in this study was used to assign an enrolled dog to group 1 (IP; Credelio Plus; MO + Lotilaner) or group 2 (CP; Sentinel Flavor Tabs; MO + lufenuron) in a 1:1 ratio in sets of two dogs. At the initial screening visit at each veterinary clinic on day −1, dogs were weighed, given a physical examination, had blood collected for hematology, blood chemistry and HW (antigen and microfilaria; see details below) testing. A designated dispenser masked to treatments at each veterinary clinic was solely responsible for dispensing the IP and CP tablets and giving and reviewing product administration instructions to each owner. The IP chewable tablets were supplied in five different strengths to provide the targeted dose range of the flavored tablet dosage form of the IP and a unit dose reflecting the intended oral treatment at approximately 20–40 mg lotilaner per kg body weight and approximately 0.75–1.5 mg milbemycin oxime per kg body weight. The CP, Sentinel Flavor Tabs (milbemycin oxime/lufenuron; Merck) were dispensed per label directions. While at the veterinary clinic, the owner was instructed on storage and administration of the assigned product in the home environment. The owner administered the tablets to the enrolled dog at home and evaluated and recorded product consumption once monthly for 11 months. IP oral tablet acceptability was evaluated in the product safety population, from acceptability data associated with the method of treatment (free choice, in food, pilling). At the day −1 visit a sufficient supply of the assigned product (IP or CP) was dispensed to the owner to last until the next scheduled visit. Following visit 1 (day −1), the owner treated the dog with the assigned product (IP or CP) a total of 11 times during the study, administered on the following targeted treatment days: 0, 30, 60, 90, 120, 150, 180, 210, 240, 270, and 300. Monthly treatments subsequent to the first treatment (day 0) were given within 30 ± 5 days after the previous treatment. Each product was given to the enrolled dog under fed conditions to ensure maximum product effectiveness.

The owner returned the dog to the clinic for various procedures and assessments on approximately days 60, 120, 180, 240, and 330. At these scheduled veterinary clinic visits, dogs were weighed and a physical exam was performed. At the initial screening visit and on days 120 and 240, each dog had blood (minimum of 1 mL for microfilaria test) collected for HW antigen and microfilaria testing. Appropriately collected blood samples from each dog were shipped overnight to a reference laboratory (IDEXX) for analysis. Blood samples collected for HW antigen were tested using the IDEXX SNAP Heartworm RT assay. The microfilarial test used was the modified Knott’s. On day 330 (study completion), dogs were weighed, given a complete physical examination, had blood collected for hematology, blood chemistry and again for adult HW antigen and microfilaria testing as described above.

### Statistical analysis

The enrolled individual dog was considered the experimental unit. For each dog, success or failure of the treatment to prevent HW infection was defined by the outcome of the HW antigen and microfilariae tests at the final day 330 visit. A positive result from either adult HW antigen test or microfilarial test confirmed with repeat testing was considered a treatment failure (termed a “positive dog”). If at least one dog, in either group, was positive at the final day 330 visit, then the non-inferiority of the combination IP compared to the CP for the prevention of HW infection was tested at a 5% non-inferiority margin using a 95% one-sided confidence interval (obtained by constructing a two-sided 90% confidence interval). The statistical package SAS 9.3 was used for analysis.

## Results

### Field study

#### Demographics and evaluable populations

**Fig. 1:**
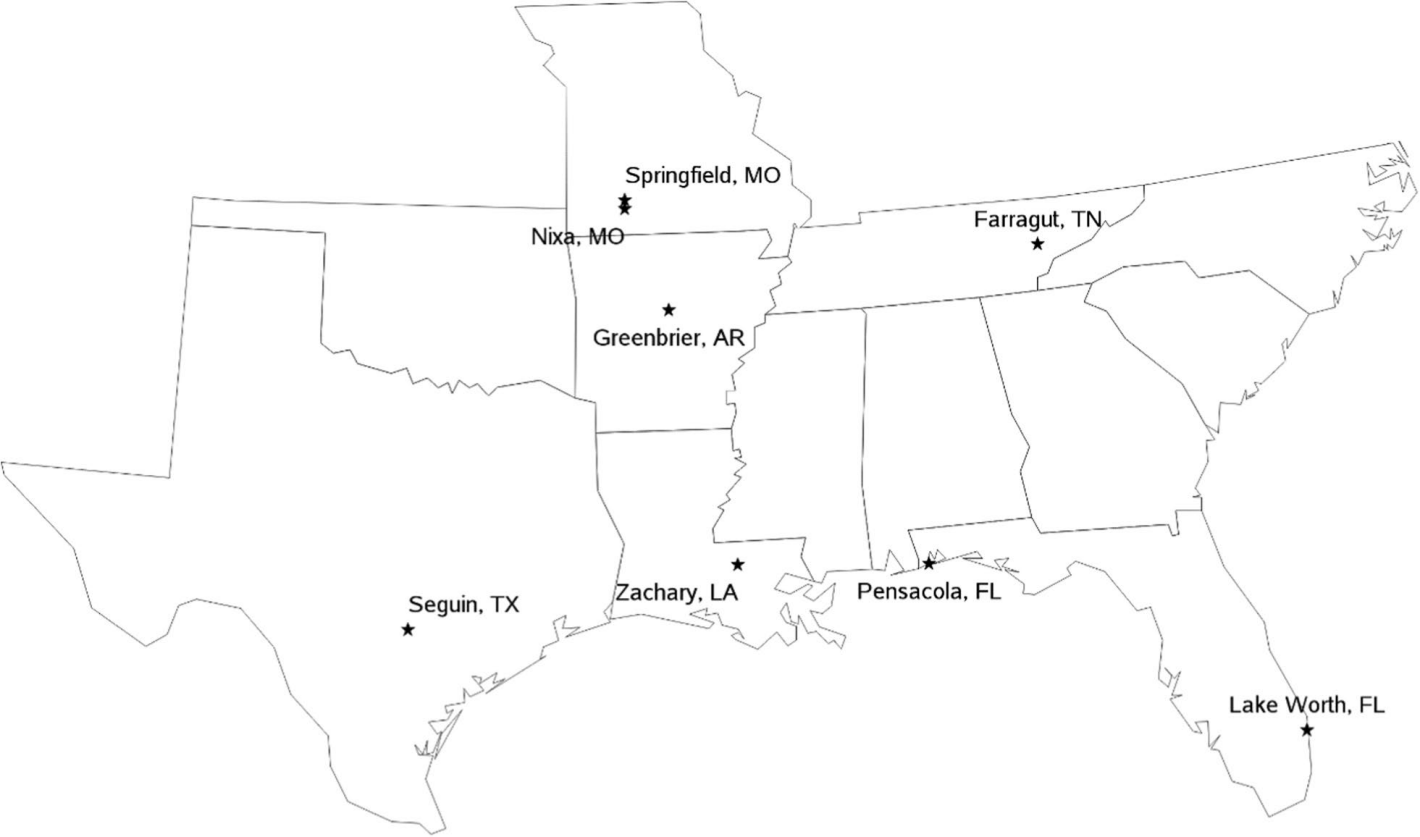
USA state locations of enrolled client-owned dogs from 8 veterinary clinics in this clinical field study investigating the heartworm prevention effectiveness and safety of a combination IP containing milbemycin oxime and lotilaner (Credelio Plus)

A total of 325 dogs were screened and recruited for the study from eight veterinary clinics throughout the United States. Veterinary clinic locations are summarized in Table 1 and shown in Fig. 1. A total of 317 dogs were enrolled, treated and subsequently used in the analyses of safety or effectiveness for the IP and CP groups during the 11-month study period. Data from all dogs receiving at least one dose of the intended IP or CP were included in the study analysis and results were summarized. Dogs in the effectiveness and safety populations were generally similar with respect to demographic conditions between the two treatment groups. The demographics of the enrolled dogs in the effectiveness and safety-evaluable populations are summarized in Tables 2 and 3, respectively. The safety population included 159 dogs in the IP group and 158 in the CP group. The effectiveness population included 112 dogs in the IP group and 126 in the CP group.

**Table 1:** Location of 8 veterinary clinics in this field study investigating the efficacy and field safety of a combination product containing milbemycin oxime and lotilaner (Credelio Plus) for the prevention of heartworm disease (*Dirofilaria immitis*) in client-owned dogs in the USA

US clinic location: city and state	Safety population: treated cases participating in the study by clinic location	
	IP	CP
Pensacola, FL	6	7
Lake Worth, FL	30	30
Farragut, TN	16	16
Springfield, MO	22	22
Seguin, TX	20	19
Greenbrier, AR	16	16
Nixa, MO	20	20
Zachary, LA	28	29
**TOTAL**	159	158

**Table 2:** Demographics of dogs enrolled as veterinary patients and included in the effectiveness-evaluable population for the prevention of heartworm disease (*Dirofilaria immitis*) in client-owned dogs in the USA

Demographic	US heartworm prevention field study	
	Credelio Plus (*n* = *112*)	Milbemycin oxime + lufenuron (*n* = *126*)
Purebred (*n*) (%)	79 (70.5)	57 (45.2)
Crossbred/mongrel (*n*) (%)	33 (29.5)	69 (54.7)
Age, mean (months)	47.8	48.0
Age, range (months)	2.0–158.0	2.0–163.0
Age group < 12 months (*n*) (%)	35 (31.3)	36 (28.6)
Age group ≥ 12 months (*n)* (%)	77 (68.8)	90 (71.4)
Male (*n*) (%)	61 (54.5)	53 (42.1)
Female (*n*) (%)	51 (45.5)	73 (57.9)
Body weight, mean (kg)	16.0	18.0
Body weight, range (kg)	2.1–50.7	2.0–61.1

**Table 3:** Demographics of dogs enrolled as veterinary patients and included in the safety-evaluable population for the prevention of heartworm disease (*Dirofilaria immitis*) in client-owned dogs in the USA

Demographic	US heartworm prevention field study	
	Credelio Plus (*n* = *159*)	Milbemycin oxime + lufenuron (*n* = *158*)
Purebred (*n*) (%)	111 (69.8)	76 (48.1)
Crossbred/mongrel (*n*) (%)	48 (30.2)	82 (51.9)
Age, mean (months)	48.6	46.8
Age, range (months)	2.0–158.0	2.0–165.0
Age group < 12 months (*n*) (%)	52 (32.7)	48 (30.4)
Age group ≥ 12 months (*n)* (%)	107 (67.3)	110 (69.6)
Male (*n*) (%)	79 (49.7)	65 (41.1)
Female (*n*) (%)	80 (50.3)	93 (58.9)
Body weight, mean (kg)	16.4	18.0
Body weight, range (kg)	2.1–50.7	2.0–61.1

In both effectiveness and safety populations, dogs were at least 8 weeks of age at the time of their enrollment and weighed at least 2 kg. Dogs ranged in age from 2 to 165 months (> 13 years). There were fewer enrolled juvenile dogs as compared to adult dogs. The proportion of female and male dogs in both populations were similar. Cross breed and pure breed dogs were enrolled in the study. In the safety and effectiveness IP population dogs, 29.5–30.2% were a cross breed and 69.8–70.5% were a pure breed (Tables 2 and 3). A variety of breeds were represented in this study, with Golden Retrievers, Labrador Retrievers, Boxers and Mixed breed dogs being the most common. Breeds commonly affected by a mutant MDR1 gene related to avermectin toxicity were also included in this field study (Australian Shepherds, Shetland Sheepdogs, English Shepherds, German Shepherds and Collie cross breeds).

#### Heartworm evaluations at day 330 (visit 6)

None of the dogs in either the IP or CP group were HW positive for either adult *D. immitis* antigen or blood microfilariae at visit 6 on day 330 (100% prevention). No formal statistical testing regarding HW prevention effectiveness was conducted, since all dogs were negative for HW at visit 6 on day 330 in both treatment groups.

#### Credelio Plus tablet acceptance

Overall, 1562 doses of IP were administered to dogs in the safety population during the study; 81.8% of doses were accepted either free choice or in food with 18.2% of doses administered by pilling. No doses were refused. A summary of tablet acceptance is presented in Table 4.

**Table 4:** Summary of tablet acceptance for dogs administered Credelio Plus tablets in the safety population for the prevention of heartworm disease (*Dirofilaria immitis*) in client-owned dogs in the USA

Treatment group	Number of dogs	Tablet consumed free choice or with small amount of food (%)	Manually dosed [pilled] (%)
Credelio Plus tablets	159	1277 (81.8%)	285 (18.2%)

#### Health observations

During the course of the 11-month study period, all abnormal events, regardless of their causality, duration, or severity, were recorded for each enrolled dog. The majority of these abnormal health events were categorized as non-serious and were not related to treatment with either IP or CP. The most common events recorded in both treatment groups were diarrhea and vomiting. Adverse events that occurred in 2.0% or more of treated dogs in one or both treatment groups also included lethargy, anorexia, and pruritus. The recorded abnormal observations that occurred were similar in frequency in both treatment groups. Serious adverse events were documented for 14 dogs (8 IP dogs, 6 CP dogs). In the IP group, serious adverse events included intervertebral disc disease in three dogs, vehicular accidents in two dogs, sepsis secondary to aspiration pneumonia in one dog, heat stroke in one dog, and vomiting with bloody diarrhea in one dog. In the CP group, serious adverse events included vehicular accidents in two dogs, neoplasia in two dogs, death due to possible poison ingestion in one dog, and renal insufficiency with regenerative anemia in one dog. The study site investigators assessed most of these observed serious adverse events as unrelated to the IP or CP treatments based on clinical examinations, history, and timing of each event.

Post-treatment hematology and serum chemistry were similar between the treatment groups and were generally within the normal reference range. For those out-of-range values, they were generally present in individual dogs at the beginning of the study, remained stable and did not increase over the course of the study, and the investigator did not consider them to be clinically relevant at the final day 330 visit or could be attributed to a pre-existing condition.

The mean weight in the safety-evaluable population at visit 1 was 16.38 kg in the IP group and 17.96 kg in the CP group. At the end of study, visit 6, the mean weights were 19.78 kg and 20.39 kg for the IP and CP groups, respectively. The mean body weight increase from visit 1 to visit 6 was 4.07 kg (range: −3.13 to 36.02 kg) in the IP group and 2.23 kg (range: −10.30 to 26.40 kg) in the CP group, corresponding to 44.7% and 32.2% increases, respectively. The body weight profiles in both groups changed at similar rates over the 11-month study and were similar, including juvenile versus adult dog comparisons, based on their pre-treatment visit 1 body weights.

More than 100 concurrent treatments were given to dogs in both the IP and CP groups. The most frequently administered concurrent treatments in both groups were vaccinations. The most administered non-vaccination treatments were carprofen (18.2% of IP dogs, 18.4% of CP dogs) and isoflurane (16.4% of IP dogs and 13.9% of CP dogs). Treatments were generally administered at similar frequencies in both groups. Concurrent treatments used during the study included FDA-approved animal drugs, human drugs used off-label, alternative/herbal remedies, medicated shampoos or other topical treatments, and prescription diets. There were no adverse events associated with the concomitant use of these treatments. The IP and CP were well tolerated and used safely with numerous other treatments and vaccines routinely administered to dogs in veterinary medicine.

## Discussion

It has been established that all currently marketed ML-containing HW preventives evaluated against certain field isolates in the USA have confirmed resistance at the phenotypic or genotypic level [3, 8–10, 12, 23]. Many of these isolates are from dogs that resided or originated from the southeastern USA, including the geographic area that has been termed the lower Mississippi river valley. Documented LOE cases have been shown to be predominately related to lack of owner compliance and not due to resistance to the HW preventives containing different ML [11]. Under good owner compliance and consistent monthly dosing for 11 months, the use of MO + lotilaner (Credelio Plus) as assessed in this field study provided 100% prevention of HW disease in 112 enrolled dogs in the effectiveness-evaluable population. As summarized in other manuscripts that are part of this collection of publications on Credelio Plus, this new broad-spectrum combination treatment option of MO + lotilaner for use in dogs offers control of the most common intestinal nematodes, HW and lungworm (*A. vasorum*) prevention, and flea and tick prevention and control. This convenient combination tablet for oral administration by pet owners to their dogs will contribute to owner compliance and help to address the treatment recommendations from global veterinary practice guidelines (e.g. AHS, ESCCAP, CAPC) to prevent HW disease and other important zoonotic parasites such as *T. canis*.

The 100% prevention rate for MO as seen in this field study is similar to historical published data for other MO-containing combination drug products (Trifexis^®^; Elanco Animal Health) where 100% prevention was documented in a field study conducted for 6 months in the USA; however, the dose level of MO was lower (0.5 to 1.0 mg/kg dose range) compared to the IP combination product containing MO used in this field study dosed at 0.75 to 1.53 mg/kg. The CP used in this study as a positive reference control also gave 100% HW prevention, and its dose range of MO is similar to Trifexis [24].

Safety and tablet acceptance were assessed in this study with over 800 doses of the IP combination product administered to enrolled dogs. In this field study, 81.8% of doses were accepted either free choice or in food. Adverse events that occurred in 2.0% or more of dogs in one or both treatment groups included diarrhea, vomiting, lethargy, anorexia, and pruritus. These recorded abnormal observations are typical of those expected to occur, are routinely seen by pet owners, are commonly seen in any general dog population and they occurred with similar frequency in both treatment groups [25, 26]. A similar AE profile was also seen with the CP (Sentinel Flavor Tabs). The health observations reported in these studies were not unexpected as the single components of the combination product have been commonly used and/or are well characterized in dogs. MO used alone or in combination with other oral parasiticides has been used safely for a number of years for intestinal nematode control and HW prevention in dogs [27]. Lotilaner as a standalone product administered for fleas and ticks has demonstrated safety for dogs under field use and laboratory conditions [28–31].

## Conclusions

In this reported field study conducted in HW endemic areas of the USA, none of the combination IP-treated dogs (Credelio Plus) tested positive for adult HW infection when dosed monthly for 11 consecutive months, thus providing 100% prevention of HW disease. This multi-site clinical study using client-owned dogs demonstrated that monthly use of the flavored chewable tablets containing a combination of milbemycin oxime and lotilaner administered orally under end use conditions was safe and effective for dogs. The combination IP was successfully administered to all enrolled dogs and the recorded tablet acceptance was > 80% when offered free choice or in food.

## Data Availability

The dataset summarizing and supporting the conclusions of this article are included within the article. Due to commercial confidentiality of the research, data not included in the manuscript can only be made available to bona fide researchers subject to a fully executed non-disclosure agreement.

## Abbreviations

AHS: American Heartworm Society
CAPC: Companion Animal Parasite Council
CP: Control product
ESCCAP: European Scientific Counsel Companion Animal Parasites
HW: Heartworm
IP: Investigational product
LOE: Lack of effectiveness
ML: Macrocyclic lactone
MO: Milbemycin oxime
